# The impact of the Ebola epidemics on children’s rights: a scoping review

**DOI:** 10.1080/16549716.2022.2061240

**Published:** 2022-05-04

**Authors:** Irene Lunghi, Awa Babington-Ashaye, Jean-Dominique Vassalli, Yvon Heller, Pierre-André Michaud, Didier Wernli, Olivia Heller, Antoine Flahault, Stéphanie Dagron

**Affiliations:** a Institute of Global Health, Faculty of Medicine, University of Geneva, Geneva, Switzerland; b International Institute for the rights of the child (Institut International des Droits de l’Enfant, IDE), Sion, Switzerland; c Faculty of Biology and Medecine, University of Lausanne, Lausanne, Switzerland; d Global Studies Institute, University of Geneva, Geneva, Switzerland; e Department of Primary Care, Division of Tropical and Humanitarian Medicine, Geneva University Hospitals, Geneva, Switzerland; f Faculties of Law and Medicine, Global Studies Institute, University of Geneva, Geneva, Switzerland

**Keywords:** Africa, rights of the child, violence, right to education, right to health, public health crisis, infectious diseases outbreaks

## Abstract

**Background:**

Th**e** Ebola virus is known as one of the deadliest pathogens to infect humans. Children represent a minority of Ebola Virus Disease cases globally. Yet, the different Ebola outbreaks in Africa had a wide impact on children’s lives and children’ rights.

**Objective:**

Review the published literature to date on Children’s rights during Ebola outbreaks. Outcomes shall contribute to get a better understanding of the main limitations or violations of children’s rights, identify potential gaps in the literature and support the promotion and protection of children’s rights for current and future health crisis.

**Methods:**

A scoping review from PubMed, Medline, Cochrane Library and Web of Science was performed using PRISMA-ScR guidelines. Articles, reports and editorial, published on Ebola Outbreaks between 1976 and 2020 were retrieved. The UNCRC clusters of rights and treaty specific guidelines were used as a framework. Documents were found through a targeted search of websites from international or regional organisations involved in Ebola crises and children’s protection.

**Results:**

48 articles and reports were reviewed. Few documents focused solely on children’s rights. Several articles covered the topic of children and Ebola outbreaks. Most of the data are linked to basic health, education, discrimination of orphans and survivors. 31% of the reviewed articles underline the violence against the children (rape, abuse, Female genital mutilations), while 21% focus on the right to education. 23% cover the topic of orphans. Impact on mental health and SRH were amongst the other covered topics.

**Conclusion:**

A lack of data on children’s rights and their violations during epidemics is observed. Regional and international collaboration is needed to document the situation of children in health emergencies. Health measures and strategies based on children’s opinions and raising awareness of their crucial role in society is key. Child-centred guidelines should be developed based on these elements.

## Background

Since its discovery in 1976, the Ebola Virus (EBOV) has emerged periodically, causing more than 20 outbreaks [1,2]. The most important outbreaks in terms of cases and deaths occurred in West Africa (Guinea, Liberia and Sierra Leone) in 2013–16, followed by the Democratic Republic of Congo (DRC) epidemic in 2018–20 [2–4]. Interestingly, even with more than 40 years of hindsight, it remains difficult to forecast the spread of Ebola Virus Disease (EVD) outbreaks in Africa [5].

Importantly, some studies underline the paucity of data and limited evidence on EVD among children, especially on disease severity and prognosis. Africa has the world’s youngest population, with a median age of 18 years [6]. Despite this fact, children typically represent a minority among all the reported confirmed and probable EVD cases, ranging from 9% to 26% [1,7,8]. The findings from the West African outbreak 2013–16, together with those of previous epidemics, may indicate that children, adolescents and young adults have lower fatality rates from EVD, compared with older adults, with the exception of new-borns [1,9,10].

The consequences of the Ebola outbreaks and the outbreak responses are severe and complex [11]. The impact on children’s lives and well-being is particularly devastating, e.g. increase in child kidnappings, recruitment in the army, child marriages and orphaned children [1,7–10,12]. This observation stresses the need to protect children from a wide-range of impacts.

The conditions of children’s protection in case of emergencies is not well understood [13], especially in the event of large scale infectious diseases outbreaks, which is the case with the recent SARS-CoV-2 pandemic. The key learnings on children’s protection from the Ebola crises should bring additional data to support the related COVID-19 response on that matter. Indeed, key similarities between the current pandemic and Ebola epidemics can be found mainly in the disruptive impact on children’s protection, respect of their rights and their well-being [14].

Children’s rights provide a legal framework to protect children worldwide. The UN Convention on the Rights of the Child (UNCRC), the guidelines for the treaty-specific periodic reports to be submitted by UNCRC States parties and the General Comments developed by the UN Committee on the rights of the Child (CRC), in charge of the implementation of the Convention, were used as the main framework to conduct this research. This choice was made despite the fact that Africa is the first continent with a region-specific child rights instrument, the African Charter on the Rights and Welfare of the Child (ACRWC) adopted in 1990 by the Organisation of African Unity. This Charter has been ratified by all except five State members of the Organisation (Morocco, Sahrawi Arab Democratic Republic, Somalia, South Sudan, Tunisia) [15]. However, firstly, the African Charter remains until today underutilized in comparison to the UNCRC for the promotion and protection of children’s rights in Africa [16]. Secondly, the Charter builds on the same basic principles as the UNCRC and the differences in terms of the catalogue of rights are not substantial, with the exception of specific contexts mentioned such as Apartheid and armed conflicts. As the most important legal treaty in terms of children’s rights, and the most widely ratified human rights treaty (all UN Member States except one (USA) being State Parties to the Convention to date) [17], the UNCRC is still the main framework to be referred to. It consists of 54 articles and is divided into three parts. While parts two and three are related to the implementation of the Convention, the role of the CRC in the protection of children’s rights to human dignity and the conditions for adhesion, part one contains the legal definition of a child, a catalogue of the civil, political, economic and cultural rights of every child, and emphasizes the following four general principles to be taken into account by states for any actions concerning children: non-discrimination, best interests of the child, respect for the child’s evolving capacities and respect for his or her right to be heard.

The scoping review methodology was found as the most relevant methodology for our study as i) we did not aim to assess the quality of the published studies but rather to map evidence regarding the impact of the Ebola outbreak on Children’s rights and guide future mitigation strategies in crisis such as the COVID-19 pandemic; ii) we considered important to gather a broad range of data sources not limited to scientific publications (e.g. reports, guidelines, grey literature etc.); iii) our study had to take into consideration various findings from a body of knowledge heterogeneous in sample size, methodology and primary outcomes [18].

In this scoping review, we aim to provide a comprehensive overview and analysis of the literature published to date on the topic of Children’s Rights and in the context of Ebola outbreaks in order to better understand the extent of the human rights issues at stake and also to identify the areas of focus and improvements for present and future public health crisis such as the COVID-19 pandemic.

## Methods

A scoping review of peer-reviewed and grey literature related to the consequences of the Ebola outbreaks on Children and their rights was conducted. In accordance with Art.1 of the UNCRC, we considered a child ‘every human being below the age of 18 years unless under the law applicable to the child, majority is attained earlier’.

## Search strategy and selection criteria

Findings were retrieved from a structured search of biomedical database, the Web and references of reviewed articles.

A first search of biomedical databases (PubMed, Embase, Medline, Cochrane Library, Web of Science) focusing on the articles’ titles was conducted. The following keywords had to be present in the title: child* AND right* OR need* AND Ebola* AND Ebolavirus* OR Ebola Virus Disease OR West African epidemic*. Articles had to be written in English, French, Italian or German. We searched for articles, reports or editorial, published between 1976 and 2020, to cover all documented Ebola outbreaks and not limiting the research to the most important outbreaks in recent years.

**Figure 1.**
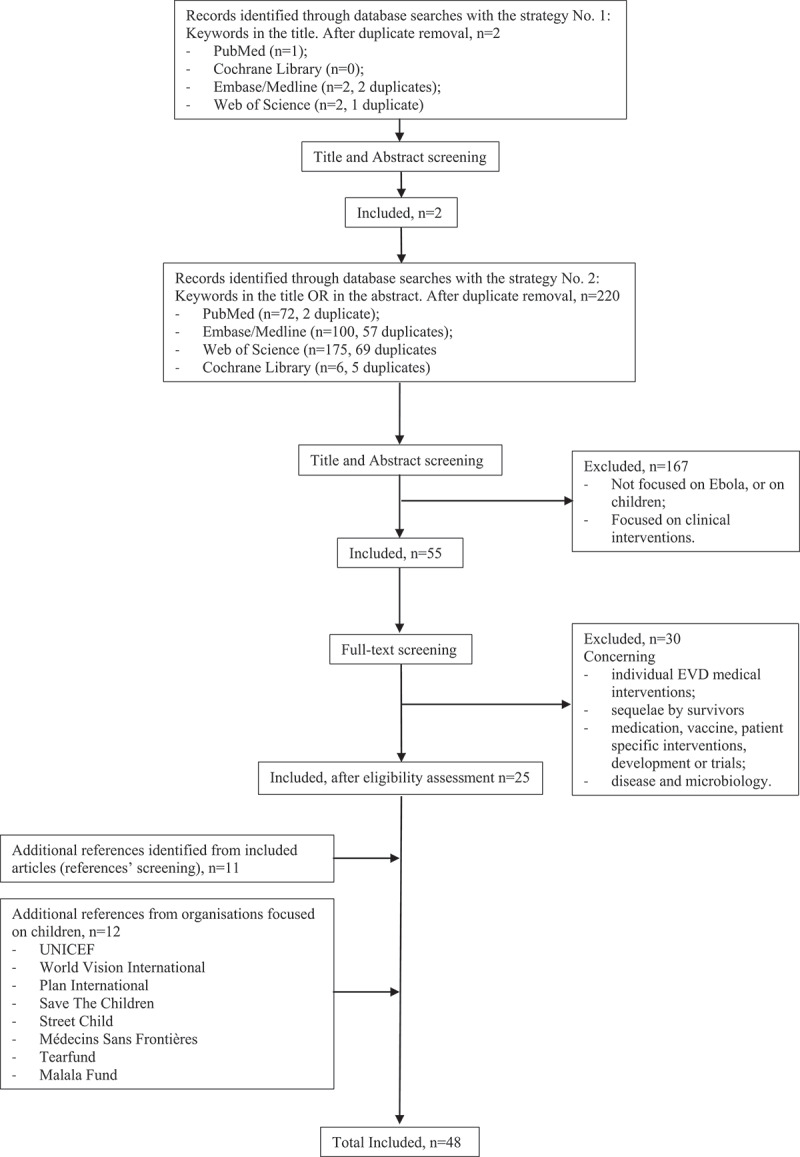
Detailed search and selection strategy (PRISMA Chart).

The scope was then broadened through a second search, including the abstracts in the search field. The last search was conducted on 02.01.2021. Search methods and selection process are summarized in Figure 1.

Research of grey literature included experts’ advice and screening for reports from organizations’ websites engaged in Ebola outbreaks responses, focusing on Children’s Rights and Welfare (extensive list summarized in Figure 1). Other international or regional organisations and organs, as well as Non-Governmental Organizations (NGOs), in charge respectively of the protection and promotion of human rights (UNHCR, ACHPR, IHRDA), and more specifically children’s rights (ACERWC, Terre des Hommes), public health (WHO, UNFPA), humanitarian action (ICRC) or peace and security (UNGA), were screened, fulfilling the research criteria, but no focused reports were found.

In order to find potential additional sources, a screening of reviewed articles’ references lists was undertaken, and further articles were identified.

Inclusion and exclusion criteria were developed to identify literature relevant for consideration within the review. The criteria were defined on the basis of the UNCRC and the clusters of rights listed in the treaty-specific guidelines regarding the form and content of periodic reports to be submitted by UNCRC States parties [19]. More specifically, the inclusion criteria were: the direct impacts of disease on children (morbidity and mortality, survival, orphanhood); and/or the impacts of EVD outbreaks and their resulting regional and international implications on civil rights and freedoms, violence, family environment, access to basic health services (immunization services, maternal care, management of common diseases such as malaria, malnutrition/undernutrition, diarrhoea, AIDS), birth and death reporting/registration, education, child labour.

## Data extraction

For data extraction, the main researcher IL used an Excel spreadsheet to complete the following information concerning the reviewed records: Title, Author(s), Date of publication, Study location focus, Type of document, Main topic, Objective(s), Population. After the literature search, all the references were imported to EndNote X9. The other co-authors screened studies for eligibility and relevance.

The information found in the reviewed articles was then classified in a table (See Table 1) according to the rights listed in the UNCRC. The same rights are then grouped in clusters and later incorporated in the guidelines for the five-year reports of the States parties.

**Table 1.** Information found in the reviewed articles related to the specific clusters of rights established by the committee on the rights of the child, used as guidelines for the treaty-specific periodic reports to be submitted by UNCRC States parties (as established by the Art. 44, paragraph 1 (b), UNCRC)

Clusters of Rights	Contents	In the context of Ebola outbreaks (according to the reviewed articles)
Civil rights and freedoms	Birth registration (Art. 7)	
	Right to identity (Art. 8)	
	Freedoms of expression (Art. 13), of thought, conscience, and religion (Art. 14), of association (Art. 15)	
	Right to privacy (Art. 16)	Lack of privacy [64].
	Right to find information from a variety of media sources (Art. 17)	Difficult access to information; [55,64–67,75] Delayed development of correct and consistent messaging about infant feeding and nutrition programming [20].
Violence against children	Protection from violence, abuse and neglect (Art. 19)	Increased abuse of boys and girls [56,57,64].
	Protection from all forms of harmful practices. Including FGM, early and forced marriages (Art. 24, para. 3)	General increase in violence on girls (rape, early and/or forced marriage, teenage pregnancy, prostitution); [12, 21–24, 55, 56, 58, 64, 65, 67, 68] Temporary decrease in FGM [55,64].
	Protection from sexual exploitation and abuse (Art. 34)	Increase in recorded cases of sexual penetration of a minor and domestic violence [68].
	Protection from cruel treatment (Art. 37)	
	Support in recovery from trauma and reintegration (Art. 39)	Challenges in finding a safe environment for EVD children survivors, often unaccompanied and/or orphans; [25,26] Disruption of medical and justice services for survivors of Sexual and Gender based Violence (SGBV) [68].
Family environment and alternative care	Parental guidance (Art.5)	
	Separation from parents (Art.9)	Orphans (Data, protection and care). [12,24,26–28,55,57,59,65,69,70]
	Family reunification (Art. 10)	Increase in family separation [64,75].
	Parental and state responsibilities (Art. 18)	
	Provide alternative care (Art. 20)	
	Adoption (Art. 21)	
	Regular reviews of treatment and situation of children receiving healthcare away from home (Art. 25)	Challenges in the provision of care to unaccompanied children EVD suspected/confirmed [25,29].
	Protection from abduction, sale and trafficking (Art. 11, 35)	
Disability, Basic Health and Welfare	Right to the best possible health and to healthcare (Art. 24)	Disrupted health service access; [27,30–38,57] except for some rural districts less affected by Ebola, utilization of primary care remained robust despite the outbreak; [39] Unavailability of medication; [30,65] Worsening of children and maternal health care quality; [26,31,33,35–37,40–43,55,65] Lack of child-centered health services; [25,27,30,40] High EVD mortality rate, especially in very young children (<12 months of age); [25,30] Exacerbation in overall infant, child and maternal mortality (specifically in teenage girls); [25,26,30,35,42,43,55,57,64] Worsening of common diseases’ management; [27,28,34,40,44,45,55,64,65,75] Increased risk of malnutrition and undernutrition; [55,57,65,75] Decline in health facility and community malnutrition screening programmes [46]. Stop of school feeding programmes; [57] Serious psychological impacts; [22,26,55,64,65,75] Failure in immunization services; [26,27,32,34,35,40,41,47–50,55,57,65,75] Disrupted SRH and family planning services [35,56,58,64].
	Rights, protection and support to children with disabilities (Art. 23)	
	Protection from drug abuse (Art. 33)	
	Right to social security (Art. 26)	Lack of assistance, misallocation of aid; [65] Erosion of safety nets and government and NGO services [21,55,64,65].
	Right to adequate and decent standard of living (Art. 27)	Worsening of poverty; [27,28,55,59,64,65,75] Increases in economic and social vulnerability [26].
Education, Leisure and Cultural activities	Right to education (Art. 28)	School closure and challenges in alternative learning; [24,55,57,64–66] Difficulties in school reopening and in students’ reenrollment [21,22,27,55,64,67].
	Education role in children’s development, the aims of education with reference also to its quality (Art. 29)	Importance of school roles and meaning in the context of an epidemic/emergency; [56,57,65,67] Insufficient level of teachers, instructors and infrastructure preparedness; [22,51] Worsening of students’ performances and grades, loss or postponement of exams [55,66].
	Cultural rights of children belonging to indigenous and minority groups (Art. 30)	
	Right to relax, play, take part in cultural and artistic activities (Art. 31)	Negative impact of social/physical distancing measures (isolation, changing in playing habits, culture and behaviours). [55,64–66]
Special protection and support measures for each of the following groups	Unaccompanied asylum-seeking children, internally displaced children, migrant children and children affected by migration (Art. 22)	
	Children belonging to a minority or an indigenous group (Art. 30)	
	Children in street situations	Increase number of children living in the street [70].
	Children in situation of exploitation, child labour (Art. 32)	Increase of children’s domestic responsibility, exploitation and child labour [22,55,57,64,65].
	Children in detention (Art. 37) and Juvenile justice (Art. 40)	Higher risk for boys to come into conflict with the law; [55] Inadequacy of legal system’s responses in addressing cases of SGBV [24].
	Children in armed conflict (Art. 38)	Increase in child recruitment in military operation in North Kivu [12].

## Results

The main findings of the conducted research to understand the literature published so far on the topic of children’s rights and Ebola outbreaks are summarized in this section. However, given the methodology used and data available, it appeared difficult to draw substantial firm conclusions about the most common violations. The hypotheses about the impact of Ebola outbreaks on children’s rights were formulated based on the number and content of the documents reviewed.

The issue of Ebola and Children’s rights was tackled from the perspective of different stakeholders such as Health professionals, NGOs and governmental actors but also educators whose expressed views were found in the literature. The impacts of the Ebola outbreaks on access to health care and education were the main subjects covered. Organizations that contributed the most to this topic include Save the Children, UNICEF and Plan International.

As mentioned in Figure 1, the first search that solely focused on key words used in the articles’ titles found 2 records in the visited databases. The search was then broadened to the abstracts and, after duplicate removal, a total of 222 articles were found through database searches. Following the application of inclusion and exclusion criteria, 25 articles were retained. 11 additional records from references screening and 12 reports from international and regional organizations and NGOs focusing on children were included (see methods), for a total of 48 documents.

Out of the 48 articles included in the study, 26 are qualitative and descriptive, including 21 organizational reports and 5 editorials. The remaining 22 records include 19 cross-sectional studies, 2 case studies and 1 observational cohort study. There is a good balance between qualitative and quantitative studies (see Supplementary Tables 1 & 2).

The vast majority of records’ reported data referred to the 2013–16 West African Ebola outbreak, with only 4 references reporting on the 2018–20 DRC Ebola epidemic. The sources focused on different countries with variable proportions: 23 on Sierra Leone, 5 on Liberia, 4 on Guinea, 4 on DRC, 1 on Nigeria. 2 reports focused on both Sierra Leone and Liberia, 8 on the three West African countries hit by the 2013–16 epidemic and 1 reporting information from different countries worldwide, including West Africa and DRC.

The time of publication covered mainly data from 2014 to 2020. The lists of all reviewed documents are included in Supplementary Table 1 & Supplementary Table 2.

In Table 1 & Table 2, we have identified the main impacts relating to the rights’ clusters and general principles defined by the CRC (and further details about this classification and rationale can be found in Supplementary Table 3). The majority of the reviewed articles, 30 out of 48 (63%), reported and analyzed the right to the best possible health and health services, underlining the exacerbation in overall infant, child and maternal mortality, the disruption of access to health services, immunization, and Sexual and Reproductive Health (SRH) services, as well as the serious psychological impacts of EVD outbreaks. The second most investigated topic is the violence against children (including rape, early and forced marriage, Female Genital Mutilation (FGM), teenage pregnancy, prostitution), described in 15 of the 48 (31%) records. Finally, 10 out of 48 (21%), articles focused on the right to education. No report or article that considered the following rights were found: birth registration; right to identity; freedom: of expression, thought, conscience, religion, and association; parental and state responsibilities; adoption; protection from abduction, sale and trafficking, and drug abuse; rights to protection and support for children: with disabilities, belonging to indigenous and minority groups, or affected by migration.

**Table 2.** Information found in the reviewed articles related to the four general principles included in the UNCRC, meant to help with the interpretation of the convention as a whole and thereby guide national programmes of implementation

General principles	Contents	In the context of Ebola outbreaks (according to the reviewed articles)
Non-discrimination (Art. 2)	Prevent discrimination and ensure that children in disadvantaged situations are able to enjoy and exercise their rights.	Profiles of children that are the most discriminated against: EVD survivors and their relatives; [12,24,26,27,40,55,64–66,69,70,75] Children from quarantined household and/or with relatives affected by EVD; [26] EVD orphans; [12,26] Pregnant girls and women [26,35,43,52,56].
		Areas of discrimination: Access to basic needs, such as water, food, shelters; [27,40,46,55,64,65] Access to school and educational programs; [21,22,27,28,55,58,64–67,75] Social exclusion and stigmatization; [12,27,59,66,69,70,75] Deprivation of a family environment; [12,27,28,59,65,69,70,75] Exploitation, abuse and children labor; [12,23,55,56,64–67,70,75] Exacerbation of gender inequalities, women and girls were among the most vulnerable. [21–23,56]
Best interests of the child (Art.3)	‘In all actions concerning children, whether undertaken by public or private social welfare institutions, courts of law, administrative authorities or legislative bodies, the best interests of the child shall be a primary consideration.’	Lower level of child protection [55,65,75].
The right to life, survival and development (Art. 6)	‘ensure to the maximum extent possible the survival and development of the child.’ Particular attention should be taken to: death registration and extrajudicial killings of children, prevention of child suicide and eradication of infanticide, no capital punishment for persons under 18 years.	Shutdown of development opportunities; [55,58] Lack of death registration [32,42,53].
Respect for children’s views (Art. 12)	Right of the child to be heard, ‘in any in any judicial and administrative proceedings affecting the child, either directly, or through a representative or an appropriate body, in a manner consistent with the procedural rules of national law.’	Children views and opinion not taken into account [55,64].

## Discussion

This scoping review contributes to a better understanding of the impact of Ebola outbreaks on Children’s rights and underlines critical issues related to the respect of fundamental children’s rights guaranteed by the UNCRC. Both the paucity of articles and our analysis of the few articles and reports found reveal the following three central findings:

Firstly, our research underscores that Children’s rights are severely and broadly impacted during Ebola outbreaks. This finding is consistent across all the countries for which scientific literature was found. Indeed, the consequences of Ebola outbreaks surpass the direct health consequences of the disease as numerous aspects of the daily life of populations are impacted. The domains found to be particularly affected are basic health care and services, education, social structure (defined as ‘the distinctive, stable arrangement of institutions whereby human beings in a society interact and live together’) [54] and the mechanisms that govern it (see Table 1 and Table 2).

Secondly, we also observe long-term effects of the outbreaks, inducing the non-respect of fundamental children’s rights (see Table 1) with a correlated and noticeable impact on the life of the future adults and on the society as a whole [55–58]. Eleven out of 48 articles (23%) reviewed addressed the topic of orphans and Ebola outbreaks. Orphanhood is acknowledged as one of the main devastating consequences of EVD on families (see Table 1, Family environment and alternative care). However, as Evans et al. pointed out, the issue of orphans in these countries has been around for a long time and the number of Ebola orphans represents only a small percentage (1.4%) of the total 702,000 orphans in the same countries [59].

Thirdly, we could not find sufficient or reliable information about the protection of children with disabilities, birth registration and right to identity, and parental and states responsibilities. As reported by UNICEF, the births of more than 70,000 Liberian children were not registered during the Ebola epidemic. UNICEF stresses the importance of birth registration as a starting point for respecting and protecting the rights of each individual [60]. Therefore, the absence of articles cannot be interpreted as reflecting the absence of rights’ violations. It rather underlines a lack of information, interest and knowledge on Children’s Rights and States parties’ obligations, representing a source of concern for the whole society. Few reviewed articles touched on the four rights defined by the CRC as part of the essential ‘principles and premises for realizing children’s right to health’ in its General Comments n° 3 and n° 15 concerning i) HIV/AIDS and the rights of the Child [61], and ii) the right of the child to the enjoyment of the highest attainable standard of health [62].

It is important to note that a large part of the available data on children’s rights and Ebola crises was collected in Sierra Leone, whereas the other countries seem to attract little attention from researchers and NGOs. The historical and significant presence of the UK in the response to emerging infections such as EVD in countries like Sierra Leone could explain the higher coverage of the situation on the field for that country. For instance, PHE (Public Health England) coordinated the UK Public Health response at national and international levels, and other bilateral partnerships and other British NGOs were involved (UK branches of Médecins du Monde and Médecins Sans Frontières) – thereby allowing a wider attention to the situation in Sierra Leone. The lack of coverage and the absence of some key sources of information (governmental and non-governmental organizations, national health systems, universities and researchers) contribute to a worsening of the data situation, whereby the quantity of data available in several countries is also observed to be negligible or non-existent. We could argue that the weaknesses of the national health and social systems generally represent important barriers to collecting data.

Based on the findings of the research, and particularly the observations of the noticeable impact on Children’s rights, it is important to reflect on potential ways to mitigate these situations. In alignment with the UNCRC and selected recommendations from organizations active in child protection (such as UNICEF, ETF, Save the Children), and thorough analysis from the research team, the following recommendations have been issued:

Firstly, general efforts should be made to fulfill, promote and protect children’s rights, which are in many cases ignored during health emergencies. For instance, through the literature review, we found that Ebola crises put children at risk of isolation, abandonment or stigmatization and all types of violence. An integrated and adapted approach to local stakeholders should be foreseen, for example ‘a family-centered support that promotes healthy family relationships, caregiver well-being and increased investment in schools and communities to ensure that every child feels safe, connected and soothed’ [63].

Furthermore, concerning the right to be heard and the right of participation, practical solutions should be established in order to have young people’s voices heard in institutional settings and during policy design, including during emergency responses. The right to be heard and have his or her views respected is a principle that is enshrined in the Convention (art. 12 of the UNCRC). Yet, only few studies asked children about their personal experiences during EVD outbreaks [55,56,58,64–70]. And so the voice of children is largely absent from reports about present and future policies that are intended to support recovery from the crisis and/or to improve the already fragile environmental context. Providing children and teenagers a voice in the development and/or in the implementation of strategies and/or laws remains unusual, and this is also observed in the current COVID-19 pandemic. This remains true even though in 2002 the CRC recommended in General Comment No. 2 the establishment of Independent National Human Rights Institutions (NHRIs) at the national level in an effort to address this issue [71]. In this document, the committee also indicates a non-exhaustive list of activities, which NHRIs should conduct, for example ‘to ensure that the impact of laws and policies on children is carefully considered’. The importance of the latter is further highlighted by a recent UNICEF and ETF report, giving voice to young people and strengthening dialogue with them in the era of COVID-19 [72], and by the International Society for Social Pediatrics and Child Health (ISSOP) commission in its global agenda for child health and wellbeing [73].

Secondly, efforts should be made to reinforce information and communication concerning the protection of children’s rights during health emergencies. On the one hand, this means ensuring the collection of reliable data throughout health emergencies, crucial to improve their monitoring. In countries affected by Ebola outbreaks, the data collection systems are relatively weak and therefore actions to strengthen them are necessary. It also appears important to implement uniform data collection tools outside emergency periods, to reinforce preparedness in times of humanitarian crises. As stated by the CRC ‘Establishing a comprehensive and reliable national data collection system [is important] in order to ensure systematic monitoring and evaluation of systems (impact analyses), services, programs and outcomes based on indicators aligned with universal standards, and adjusted for and guided by locally established goals and objectives’ (Legal analysis of Art. 19. 42. (v)) [74].

On the other hand, this means efforts to inform communities on children’s rights and how they can be protected. Prevention and awareness campaigns for the population on child-related issues are even more crucial in the context of humanitarian crises. As UNICEF pointed out, a more effective approach would seem to be to listen to the representations and concerns of communities, and to base interventions on dialogue [75]. Child protection programs should be embedded in broader interventions, including parents, families, educators, teachers, health professionals, communities, mobilizing all the various actors present in children’s daily lives. In the same article, UNICEF is calling for greater investment in mental health and psychosocial support services (MHPSS) for children in ‘fragile and humanitarian contexts’.

Thirdly, efforts should be made to reinforce the cooperation of all stakeholders in epidemics and pandemics as a relevant approach to ensure the respect of children’s rights. Our findings show that affected countries during health emergencies have very specific needs that can only be addressed through the cooperative action at the national, regional and international levels, through an integrated, whole-of-society approach. More specifically, in General Comment No. 15, the CRC provides a list of obligations and responsibilities for the involved stakeholders (UNCRC States parties, and non-State actors such as parents, caregivers, business enterprises, mass and social media, researchers, etc.) and emphasizes the importance of international cooperation [62].

The scope of these recommendations extends beyond the specific case of Ebola outbreaks and can be applied more widely to other public health crises. There are differences between the direct impact of specific infectious diseases on health. For instance, in the case of Ebola, the case fatality rate is extremely high (estimated from 40 to 78%, depending on the sources) [1,8–10], whereas for SARS-CoV-2, the case fatality rate is much lower (estimated < 0.5%) [76]. However, there were also many similarities that allow the generalization of the formulated recommendations. Indeed, and as already pointed out by The Lancet Child & Adolescent Health [77], and Save the Children [78], children’s rights have been and continue to be violated in the case of the Covid-19 pandemic, as was the case in the Ebola outbreaks. Stigmatization, emotional distress, loss of parents, violence or loss of education, are amongst the most common areas where the negative impacts of Ebola and COVID-19 can be observed. It is imperative for the protection of children that further reviews of violations of children’s rights during Ebola, COVID-19 and other health crises are made; and, most importantly, that sustainable ways be found to address them. The recently reported EVD cluster (14 February 2021) in Guinea and DR Congo indicates that the Ebola plague is still active, and thus that these concerns remain highly pertinent [79].

## Limitations

This scoping review is bound by limitations of the quantity of existing articles on Ebola and Children’s rights and/or their accessibility. The authors’ rationale for the search strategy also influenced de facto the outcomes. The databases consulted may not cover all possible reports and publications on the subject.

## Conclusion

The lessons learned and expertise from handling previous outbreaks can be used to support quick and efficient responses by different stakeholders in current and future Ebola epidemics, but also in future health crisis.

In addition, it is essential to reinforce the importance of the protection of children’s rights when new crises arise. The relevance of this scoping review findings is of utmost importance in the current pandemic context. Constant and common efforts are needed to work towards a better respect of children’s rights, with sustainable and integrated approaches understood to be important parts of the solution.

## List of abbreviations

ACHPR: African Commission on Human and Peoples’ Rights
ACERWC: African Committee of Experts on the Rights and Welfare of the Child
ACRWC: African Charter on the Rights and Welfare of the Child
AU: African Union
ICRC: International Committee of the Red Cross
CRC: Committee on the Rights of the Child
DRC: Democratic Republic of the Congo
EBOVE: bola Virus
ETF: European Training Foundation
EVDE: bola Virus Disease
FGM: Female Genital Mutilation
IHRDA: Institute for Human Rights and Development in Africa
ISSOP: International Society for Social Pediatrics and Child Health
MHPSS: Mental Health and Psychosocial Support Services
NGO: Non-Governmental Organization
SRH: Sexual and Reproductive Health
UNCRC: United Nations Convention on the Rights of the Child
UNHCR: United Nations High Commissioner for Refugees
UNFPA: United Nations Population Fund
UNGA: United Nations General Assembly
UNICEF: United Nations International Children’s Emergency Fund
WHO: World Health Organization

## Paper context

The Ebola virus disease (EVD) is a severe and often fatal disease affecting humans. Serious impacts of the Ebola outbreaks on children’s rights have been noticed. Our paper aims to review published literature on Ebola and Children’s rights; our findings highlight a paucity of data available and serious rights’ violations impairing children’s health and areas such as education, mental health, family relationships, child abuses. Promoting children’s rights and collaboration between stakeholders and countries is essential.
